# A multi-step classifier addressing cohort heterogeneity improves performance of prognostic biomarkers in three cancer types

**DOI:** 10.18632/oncotarget.13203

**Published:** 2016-08-11

**Authors:** Ellis Patrick, Sarah-Jane Schramm, John T Ormerod, Richard A Scolyer, Graham J Mann, Samuel Mueller, Jean Y.H. Yang

**Affiliations:** ^1^ School of Mathematics and Statistics, The University of Sydney, Sydney, Australia; ^2^ The Westmead Millennium Institute for Medical Research, The University of Sydney, Sydney, Australia; ^3^ Melanoma Institute Australia, The University of Sydney, Sydney, Australia; ^4^ Tissue Pathology and Diagnostic Oncology, Royal Prince Alfred Hospital, Sydney, Australia; ^5^ Discipline Pathology, Sydney Medical School, The University of Sydney, Sydney, Australia; ^6^ Program in Translational NeuroPsychiatric Genomics, Institute for the Neurosciences, Department of Neurology, Brigham and Women's Hospital, Boston, MA, USA; ^7^ Harvard Medical School, Boston, MA, USA; ^8^ Program in Medical and Population Genetics, Broad Institute, Cambridge, MA, USA; ^9^ ARC Centre of Excellence for Mathematical & Statistical Frontiers; ^10^ Program in Translational NeuroPsychiatric Genomics, Institute for the Neurosciences, Department of Psychiatry, Brigham and Women's Hospital, Boston, MA, USA

**Keywords:** biomarker, classification, cancer, pathology, prognosis

## Abstract

Cancer research continues to highlight the extensive genetic diversity that exists both between and within tumors. This intrinsic heterogeneity poses one of the central challenges to predicting patient clinical outcome and the personalization of treatments. Despite progress in some individual tumor types, it is not yet possible to prospectively, accurately classify patients by expected survival. One hypothesis proposed to explain this is that the prognostic classifiers developed to date are insufficiently sensitive and specific; however it is also possible that patients are not equally easy to classify by any given biomarker. We demonstrate in a cohort of 45 AJCC stage III melanoma patients that clinico-pathologic biomarkers can identify those patients that are most likely to be misclassified by a molecular biomarker. The process of modelling the classifiability of patients was then replicated in a cohort of 49 stage II breast cancer patients and 53 stage III colon cancer patients. A multi-step procedure incorporating this information not only improved classification accuracy but also indicated the specific clinical attributes that had made classification problematic in each cohort. These findings show that, even when cohorts are of moderate size, including features that explain the patient-specific performance of a prognostic biomarker in a classification framework can improve the modelling and estimation of survival.

## INTRODUCTION

The biological complexity of most disease states limits the performance of prognostic biomarkers [1–3]. Therefore, identifying and understanding the sources of this complexity is critical for improved prediction of prognosis. Due to the difficulties associated with classifying heterogeneous data, in many studies patients are often prospectively segregated with respect to known genetic or pathological features (e.g. pathologic tumor stage or hormone receptor status in breast cancer) that might confound interpretation before classification and data analysis. The inherent complexity of most human cancer cohorts and practical limits of identifying enough patients of a similar demographic and pathologic stage create a reality whereby partitioning patients is still not guaranteed to produce a sufficiently large homogeneous study cohort. Demonstrating this, we previously observed that there were subsets of AJCC stage III metastatic melanoma patients whose clinical outcome could be more easily explained by different clinical pathological or molecular biomarkers than others [4], an observation subsequently confirmed in breast cancer [5]. One interpretation of these results is that even after prospectively restricting study inclusion criteria to a specific pathologic stage – e.g., metastatic lymph node samples from patients with AJCC stage III disease in the melanoma study – there is a further subset of patients for whom the different clinical, pathological or molecular measurements point to alternative, and even competing, predicted outcomes for a given individual patient. The observation that patients may vary by classifiability itself opens the question of whether it is possible to identify the subset of patients most likely to be correctly classified by a given biomarker of interest, compared with those who are not, prior to the application of that biomarker.

Herein we propose one such model capable of retrospectively identifying clinical and pathological features that identify and explain which patients are more likely to be successfully separated according to a good and poor prognosis by gene expression data. By including this identification step in a classification procedure we then show that we can substantially improve the accuracy of predicting prognosis in three cancer datasets; 45 AJCC stage III melanoma patients [6], 49 stage II breast cancer patients [7] and 53 stage III colon cancer patients [8]. These three cancer datasets were chosen as for each patient they had well annotated pathological and treatment information as well as gene expression data and the cohorts maintained moderate sample sizes after restricting focus to tumour stages that had a similar number of patients with good and poor prognosis. Our proposed approach to classifying these patients not only improves the prediction of their prognosis by identifying potential sources of heterogeneity, it also helps identify the clinical or pathological features which might explain whether a patient would be more accurately classified by readily available clinical information or more cost and time sensitive gene expression data.

## RESULTS

**Figure 1:**
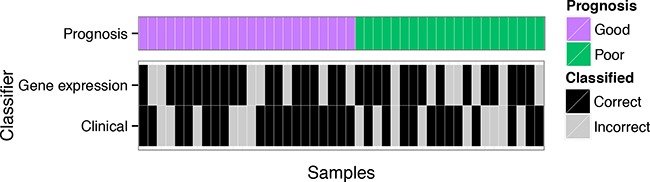
Classification errors from melanoma data The leave-one-out cross-validation errors for each patient in a melanoma cohort [6] from biomarker models built using DLDA [16] for the gene expression data and logistic regression on the clinical data are split on prognosis.

**Figure 2:**
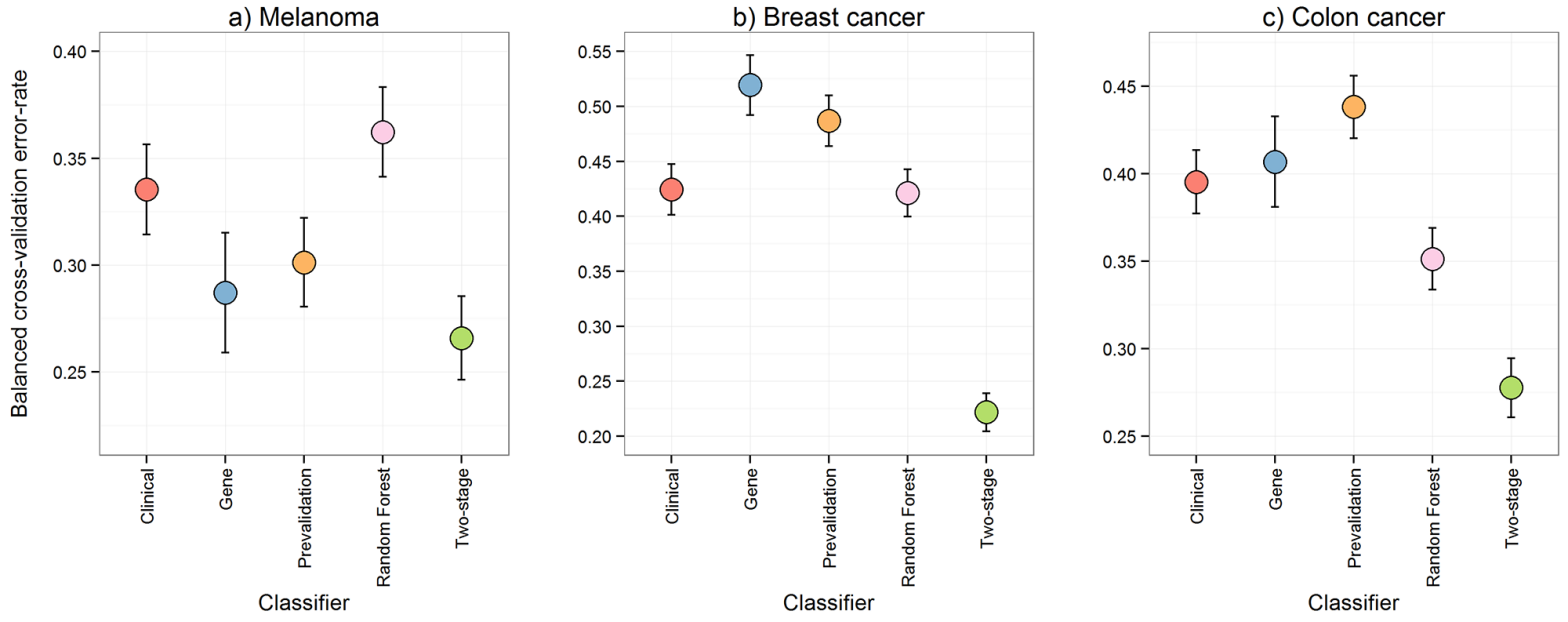
Comparison of predictive performance The leave-two-out cross-validation balanced error-rates from five methods performed on the melanoma, breast cancer, and colon cancer data were compared to assess predictive performance. The five methods were: 1) DLDA [16] on the gene expression data only; 2) logistic regression on the clinical variables only; 3) a pre-validation approach [9] for integrating the gene expression and clinical data; 4) Random Forests [10] on the gene expression and clinical data to capture interaction effects; and, 5) our proposed multi-step classification approach. Error bars represent the 95% confidence intervals.

We previously observed that there were subsets of AJCC stage III metastatic melanoma patients whose prognosis, death within a year of resection or survival for more than four years after resection, was more easily predicted than others for a variety of biomarkers [4]. This insight is demonstrated in Figure 1 which shows the classification performance at a patient level for a biomarker constructed with either gene expression data or clinico-pathologic and mutation variables (“clinical” variables) in the melanoma cohort [4]. The two data types – gene expression and clinical - classified different patients as having a good or poor prognosis with: 1) some patients classified correctly by both biomarkers; 2) patients for which only one biomarker was accurate; and, 3) patients where both failed. While the gene expression and clinical biomarkers classified the patient samples differently, they had a similar predictive performance on average. The balanced leave-two-out error-rates are given in Figure 2a where the biomarker built on gene expression alone achieved an error rate of 29% in the melanoma data while the clinical data produced an error rate of 34%.

Given that the gene expression and clinical data might be detecting different prognostic signals for different patients, two standard methods for integrating gene expression and clinical data were applied to the melanoma data and two additional cohorts in separate cancers: breast [7] and colon [8]. Figure 2 contains the balanced error-rates of these integration approaches, pre-validation (PV) [9] and random forests (RF) [10], for estimating prognosis. PV is a linear classification approach, which reduces the likelihood of the potentially many gene expression measurements swamping the relatively few clinical variables. RF is a classification approach that can handle non-linear interactions and can thus fit more complex models. In all datasets PV and RF were either inferior, or, only marginally superior to the clinical or gene expression biomarkers alone in estimating prognosis for overall or disease-free survival, with each of the integration approaches performing worse than at least one of the individual biomarkers in at least one cohort. Any actual improvement from either PV or RF was modest at best, with the exception of the RF approach in the colon cancer data.

**Figure 3:**
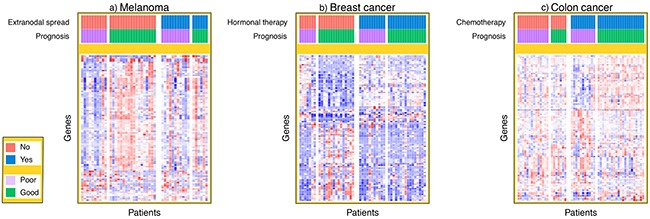
Heat maps of gene expression signal The standardized gene expression signal is plotted for the top 100 genes with largest fold change for each patient in the melanoma, breast cancer and colon cancer cohorts. In the heat map, each row represents a different gene and each column represents a different patient sample. The patients from each dataset have been ordered by both prognosis and either extranodal spread, hormonal therapy or chemotherapy in the melanoma, breast cancer and colon cancer cohorts respectively.

We then took a different approach, hypothesizing that there would be clinical variables associated with the classification accuracy of the gene expression biomarker. Extranodal spread was identified as the most informative clinical variable for accurate classification of melanoma patient outcome by the biomarker constructed with gene expression data. Figure 3a shows a heat map of expression of the 100 genes with the largest fold changes between good and poor prognosis in that cohort. We observed clear differences in the gene expression signal between patients with and without extranodal spread. Similar observations were made in the breast cancer and colon cancer cohorts; we identified hormonal therapy and adjuvant chemotherapy in those two cancers, respectively, as the most informative clinical variables for explaining the classification accuracy of the gene expression data. Figure 3b and 3c show that the gene expression data contained prognostic signal for patients without hormonal therapy and with adjuvant chemotherapy.

**Figure 4:**
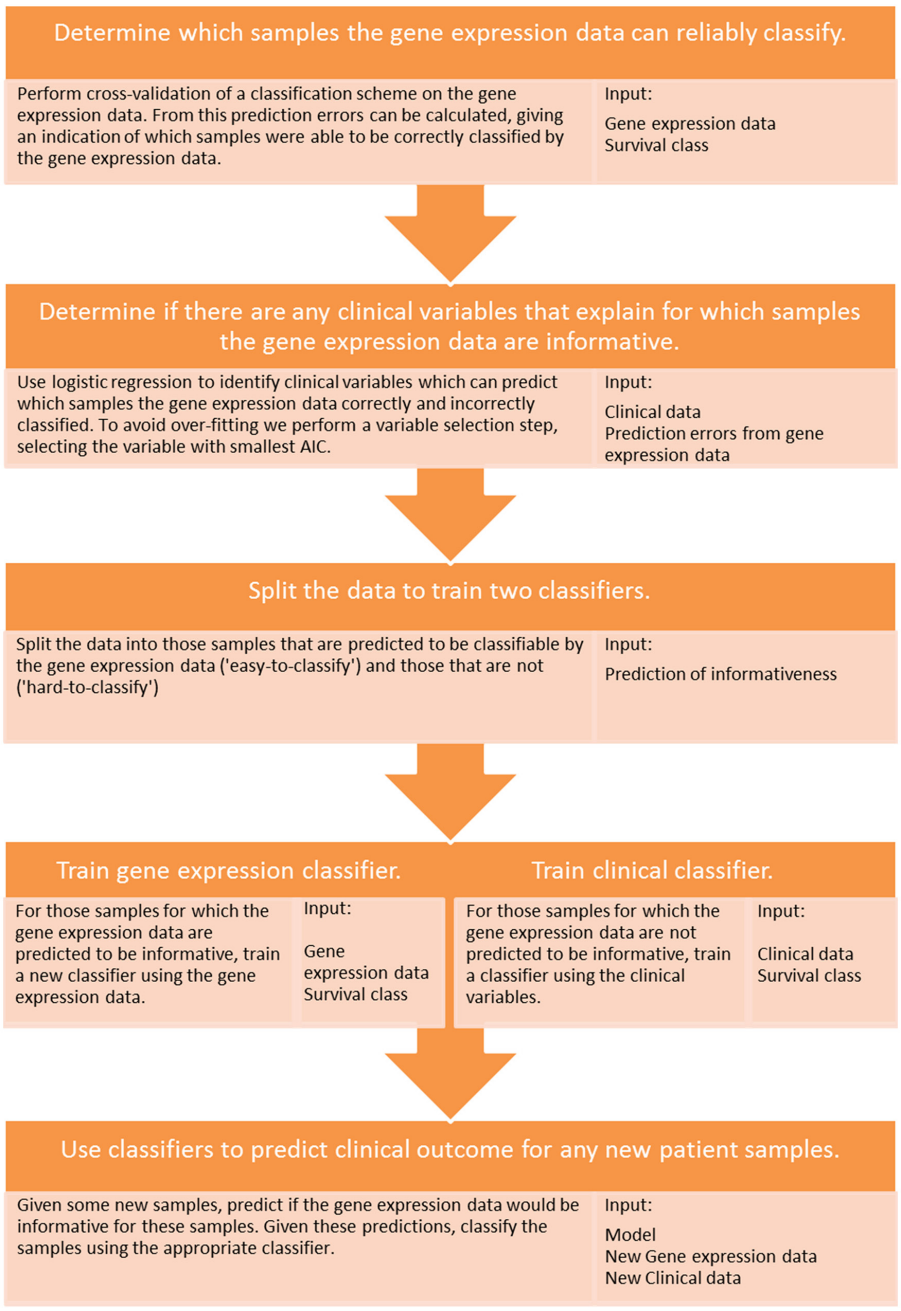
Schematic of the proposed multi-step classification approach A diagram detailing the multi-step classification approach and the input data types required at each stage of the process.

We therefore propose a new multi-step classification framework that includes modelling of gene expression biomarker performance, and which markedly improved estimation of prognosis in three different cancer types (Figure 2). First, a classifier is built using the gene expression data to predict a clinical outcome of interest. The associated clinical data are then used to determine which clinical variable is able to predict the subset of patients that can be reliably classified by the gene expression information. The cohort is then divided into ‘easy-to-classify’ and ‘hard-to-classify’ groups. The gene expression data are then used to build a classifier only for patients in the easy-to-classify group, leaving the clinical data to predict the outcome of the remaining subset of patients i.e., those identified as hard-to-classify. This procedure, which is described in further detail in the methods section and in Figure 4, led to clear reductions in patient outcome classification error rates relative to any of the aforementioned approaches as shown in Figure 2 and Supplementary Tables S2, S3 and S4. Compared with the best performing individual biomarker in each cohort our multi-step classifier had a balanced error-rate of 26% compared to 29%, in the melanoma cohort, 22% compared to 42% in the breast cancer cohort and 28% compared to 40% in the colon cancer cohort. When further contrasted to pre-validation and random forest, these improvements in classification performance are substantial given the sample sizes in these cohorts are only moderate.

**Figure 5:**
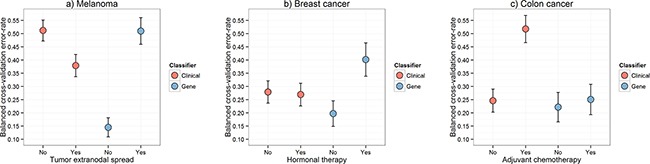
Error rates calculated within the identified classifiability sub-categories Boxplots of the leave-two-out cross-validation balanced error-rates calculated within each of the two identified classifiability sub-categories for each cancer for models built using DLDA [16] in the gene expression data and logistic regression in the clinical data in the **a.** melanoma, **b.** breast cancer and **c.** colon cancer cohorts. For the gene expression data boxplots, two completely independent cross-validated classification schemes were performed within both of the two patient groups: a) with and without tumor extranodal spread; b) with and without hormonal therapy; c) with and without adjuvant chemotherapy.

We further analyzed why classification performance in each of the three cancer cohorts could be improved by the multi-step approach. Specifically, we aimed to investigate whether there may simply be multiple signals in the data with one signal overpowering the others. Figure 5a shows the classification error rates produced after biomarkers were independently built and validated within the hard-to-classify (with extranodal spread) and within the easy-to-classify (without extranodal spread) subsets of melanoma patients for both the gene expression and clinical data. Error rates were lower using the gene expression biomarker for those patients without extranodal spread and higher for those with extranodal spread, and vice versa for the clinical data. This observation is a clear indicator of why our approach worked for this cohort of patients; a lower error rate demonstrates that a respective data source is more informative for those patients since the hard-to-classify patients still had high error rates even when a model was fit only using these patients. Similar patterns were observed in the two other cancers examined (Figure 5b and 5c).

## DISCUSSION

We have shown that by conceptualizing error rates as a measure of classifiablilty, we can identify clinical determinants of this classifiability and then leverage these determinants to construct superior classification models. Importantly, none of the key clinical determinants could have been selected a priori as most likely to confound classification. That is, pre-determining potential confounding variables before an experiment without sufficient evidence and using these to subset a cohort might have unnecessarily reduced power or further confounded the analysis [1, 11, 12]. Our results indicate that confounding variables can be identified empirically and then included within the classification model building process, with the effect of substantially reducing prognostic prediction error rates.

Our results imply that resources should be invested into both the measurement of gene expression and the collation of detailed clinical information to improve the accuracy of prognostic estimates for cancer patients. Both the clinical and gene expression data contain useful prognostic information, however, these two classes of information have different relevance to different patients. The pivotal message from Figure 5 is that the poor performance of biomarkers built on the gene expression or clinical data alone (Figure 1) is not a result of those biomarkers being unable to capture complex and multiple signals within each data level (although the gene expression data in the colon cancer cohort may perform better with either boosting [13] or more complex models [10, 14]). Instead, it appears that these single data type biomarkers justify the measuring of information at multiple molecular and phenotypic levels, as different levels of the data do appear to contain prognostic signal for different patients. The limited availability of high quality specimens with linked, well-annotated clinical and pathologic data is an ongoing challenge in many diseases including cancers [15]. Although restricted in power, studies such as the present one can make important contributions to the development of hypotheses and methodological approaches within a field, with a view to validation once larger cohorts are available.

Interestingly, standard methods for integrating gene expression and clinical data did not perform competitively on these datasets. This was surprising since, in the melanoma cohort, both the gene expression and clinical data classified different patients correctly suggesting that integrating the two would be superior. Nonetheless, the question remains: why did the two integrative approaches not provide more substantial improvements to classification performance in these three datasets? To begin with, since PV is simply a linear combination of the clinical variables and a biomarker built from the gene expression, it might not be capable of capturing the inherent complexity of the data. In contrast, RF is a non-linear classification method built using decision trees making it capable of describing complex models, but despite its use of resampling methods it may still be over-fitting to the data [11].

To conclude, we have demonstrated that by identifying clinical variables that confound gene expression biomarker performance in individual patients we were able to improve the prediction of patient clinical outcome in three cancer cohorts. The value in this finding is that it is not always clear which sources of heterogeneity might be confounding a discriminatory signal within a dataset, and this is especially true in complex diseases such as cancers. Our multi-step procedure identified the clinical variables that were most likely to obscure a potentially useful gene expression signal of interest. It therefore has strong potential to help build models of outcome that have greater translational relevance, including aiding clinicians in determining the most appropriate schedule for clinical follow up of cancer patients, through substantial improvements to their accuracy. There is also potential value in the cost-effectiveness of this approach, which begins by leveraging the clinic-pathologic information that is immediately available to the clinician in order to enhance the value of molecular biomarkers that are measured with more difficulty and at higher cost.

## MATERIALS AND METHODS

### Melanoma specimens – clinical and pathologic, and molecular data

We used the previously reported global mRNA expression profiles from 45 AJCC stage III metastatic melanomas (deposited in GEO Accession Number: GSE54467), and linked detailed clinico-pathologic data, including *BRAF* and *NRAS* mutation status, as described in [6]. Survival times were as previously analyzed [6], and we compared those same patient groups already reported: patients surviving more than four years after resection of lymph node metastatic disease with no sign of relapse (n_Melanoma_GoodPrognosis_=22), and patients who died of melanoma within 12 months of resection (n_Melanoma_PoorPrognosis_ =23). We considered the following 11 clinico-pathologic variables in our analysis: patient age (at banking), patient sex, size of nodal metastatic tumor (mm), extranodal spread (present vs. absent), number of metastatic nodes, primary melanoma cell shape (round vs. ovoid, elongated, and spindle), percentage of necrosis and degree of pigmentation. *BRAF* and *NRAS* mutation status were identified via the Sequenom OncoCarta v1.0, MelaCarta v1.0 platform followed by MassARRAY25 mass spectroscopy, as previously described [6]. These variables and patients were selected from a larger pool available to avoid either the imputation of missing values or the discarding of more patients and are shown in Supplementary Table S1. Variables with information missing for five or less patients were selected for analysis and following this all patients with any remaining missingness were removed.

### Breast cancer specimens – clinical and pathologic, and molecular data

We used previously reported global mRNA expression profiles of human breast cancer samples together with clinical history [7] (deposited in ArrayExpress, experiment number: E-TABM-158). Briefly, 83% of tumors were early stage (stage I and II) with an average diameter of 2.6 cm. Approximately half of the tumors were node positive and 67% were estrogen receptor positive. Most patients (60%) received tamoxifen and half received adjuvant chemotherapy (typically Adriamycin and Cytoxan). As with the melanoma cohort above, survival times had previously been analyzed [7]. Following on from this, and also restricting our analysis to stage II patients, we split the patients into two categories; those with recurrence less than five years (18 patients) and greater than five years (31 patients). The clinical variables used in the analysis are shown in Supplementary Table S1 and were chosen to avoid either the imputation of missing values or discarding of more patients. Variables with information missing for five or less patients were selected for analysis and following this all patients with any remaining missingness were removed.

### Colon cancer specimens – clinical and pathologic, and molecular data

We used previously reported global mRNA expression profiles of human colon cancer samples together with clinical history [8] (deposited in GEO Accession Number: GSE39582). The French national Cartes d’Identité des Tumeurs (CIT) program involves a multicenter cohort of patients with stage I to IV colon cancer who underwent surgery between 1987 and 2007 in seven centers. Patients who received preoperative chemotherapy and/or radiation therapy and those with primary rectal cancer were excluded from the study. Survival times for this study had previously been analyzed [8]. To limit heterogeneity and balance classes we restricted analysis to tumor node metastasis stage III patients and split them into two categories; those with over-all survival less than three years (25 patients) and greater than seven years (28 patients). The clinical variables used in the analysis are shown in Supplementary Table S1 and were chosen to avoid either the imputation of missing values or discarding of more patients. Variables with information missing for five or less patients were selected for analysis and following this all patients with any remaining missingness were removed.

### Patient classification using gene expression data

Classification was undertaken on the gene expression data as follows: 1) Feature selection, selecting the genes to include in a model, was performed by selecting the one thousand genes with largest fold change; 2) Using the class information, a diagonalized linear discriminant analysis (DLDA) [16] was trained on the selected genes to build a classifier. DLDA was chosen due to its wide spread use as a simple gene expression classification method [17]. To avoid an additional level of cross-validation we decided to make a fixed and arbitrary choice of using the top one thousand genes. While DLDA was chosen here for illustration, in further application of our multi-step framework any classifier can be used instead.

### Patient classification using clinico-pathologic and mutation (‘clinical’) data

Classifiers using the clinical data were built using logistic regression in conjunction with step-wise AIC variable selection. While logistic regression was chosen here for illustration, in further application of our multi-step framework any classifier can be used instead. Step-wise AIC was chosen as it is the default model selection choice for many logistic regression packages. Other choices are possible such as using step-wise methods with other information criteria [18]. However, investigating what method further improves on the gain in prediction accuracy observed in this study would distract from the main aim of this article.

### Patient classification using clinical and gene expression data combined – a pre-validation approach

We used a pre-validation approach [9] to integrate the gene expression and clinical data for prediction of patient clinical outcomes. Leave-one-out cross-validation (LOOCV) was used to construct a pre-validated decision vector for the gene expression data via DLDA [16]. As LOOCV was used, then for each sample its value in the pre-validation vector is the predictor from the prediction rule constructed using DLDA with all other samples. This pre-validated decision vector was then integrated with the clinical data after step-wise AIC variable selection using logistic regression.

### Patient classification using clinical and gene expression data combined – a random forest approach

Integration of gene expression and clinical information to predict patient clinical outcome was also performed using a random forest [10] classification scheme. Using a resampling strategy, Random forests is an approach that constructs multiple decision trees and combines these into an ensemble classifier. Unlike logistic regression, Random forests can handle highly non-linear interactions and classification boundaries. We used LOOCV to construct a pre-validated decision vector for the gene expression data using DLDA. Random forests analysis was then used to build a model using the pre-validated gene expression vector and the clinical data.

### Identifying clinical variables that predict for which samples gene expression data would be informative

Classification error rates for individual patients were calculated by performing LOOCV of a classification scheme on the gene expression data. In general, such errors could alternatively be calculated using bootstrap, k-fold cross-validation or repeated k-fold cross-validation. Logistic regression was then used, treating the prediction errors as a response and using the clinical variables as explanatory variables. The clinical variable with smallest AIC was selected to divide the patients into two cohorts; those patients who could be classified with the gene expression data (referred to herein as ‘easy-to-classify’) and those who could not (‘hard-to-classify).

### Assessing the signal within ‘easy-to-classify’ and ‘hard-to-classify’ subgroups

If a clinical variable was identified as having the potential to explain the heterogeneity in the gene expression data there may me some question as to whether the samples within any of its levels contained signal on a given platform. To test if this was the case, the samples were divided by the levels of the clinical variable (melanoma: with and without extranodal spread, breast cancer: with and without hormonal therapy, colon cancer: with and without adjuvant chemotherapy). Within these splits, a leave-2-out cross-validated classification scheme was independently performed.

### A multi-step classification approach

First, the clinical variables were used to predict which samples the gene expression data could and could not classify in the LOOCV. Using this prediction, the samples were divided into ‘easy-to-classify’ and ‘hard-to-classify’ subsets. A new classifier was built on the gene expression data for the patients in the former group while the clinical variables were used to construct a classifier for patients in the latter (i.e., where the gene expression data were predicted to be uninformative). This approach was implemented at the level of individual patients wherein for each case the clinical variable was first used to predict whether the gene expression data would be informative and then, depending on the result (hard or easy), the appropriate downstream classifier was then subsequently applied to predict an individual's prognosis class. This process is further illustrated in Figure 5. Code for running the multi-step classification approach can be found at http://www.ellispatrick.com/classifiabilitycode.

## SUPPLEMENTARY MATERIALS TABLES




