# Supplementary material for: A multi-step classifier addressing cohort heterogeneity improves performance of prognostic biomarkers in three cancer types
Source: Oncotarget. 2016 Aug 11;8(2):2807–15. doi: 10.18632/oncotarget.13203 (PMC5356843; doi:10.18632/oncotarget.13203)
Supplement: Supplementary file 1 [file oncotarget-08-2807-s001.pdf]

A multi-step classifier addressing cohort heterogeneity improves performance of prognostic biomarkers in three cancer types

SUPPLEMENTARY TABLES

Supplementary Table S1: Details of the patients and variables used in analysis for the three cancer cohorts.

See Supplementary File 1

Supplementary Table S2: Sensitivity and specificity for predicting good prognosis for melanoma cohort

Melanoma	*Gene expression*	*Clinical*	*Prevalidation*	*Random forest*	*Two stage*
Sensitivity	0.69	0.75	0.79	0.7	0.71
Specificity	0.73	0.57	0.60	0.56	0.76

Supplementary Table S3: Sensitivity and specificity for predicting good prognosis for breast cancer cohort

Breast	*Gene expression*	*Clinical*	*Prevalidation*	*Random forest*	*Two stage*
Sensitivity	0.42	0.95	0.82	0.76	0.80
Specificity	0.48	0.00	0.03	0.27	0.71

Supplementary Table S4: Sensitivity and specificity for predicting good prognosis for colon cancer cohort

Colon	*Gene expression*	*Clinical*	*Prevalidation*	*Random forest*	*Two stage*
Sensitivity	0.63	0.67	0.60	0.67	0.76
Specificity	0.54	0.53	0.51	0.62	0.68
