# Supplementary material for: A multi-step classifier addressing cohort heterogeneity improves performance of prognostic biomarkers in three cancer types
Source: Oncotarget. 2016 Aug 11;8(2):2807–15. doi: 10.18632/oncotarget.13203 (PMC5356843; doi:10.18632/oncotarget.13203)
Supplement: Supplementary file 2 [file oncotarget-08-2807-s002.docx]

**Supplementary Table S1) Details of the patients and variables used in analysis for the three cancer cohorts.**

| Cancer | Variables used | Samples used |
| --- | --- | --- |
| Melanoma ([6](#_ENREF_6)) | Person_Sex, Age_Analysis, Tum_NumNodesInv, Tum_MetSize, Tum_Extranodal, Tum_CellType, Tum_CellSize, Tum_Necrosis, Tum_Pigment, Tum_BRAFmut, Tum_NRASmut | TB36, TB1, TB237, TB125, TB30, TB47, TB13, TB119, TB52, TB93, TB102, TB142, TB234, TB61, TB83, TB322, TB343, TB360, TB334, TB340, TB351, TB172, TB249, TB288, TB16, TB17, TB28, TB217, TB45, TB103, TB32, TB37, TB74, TB385, TB51, TB66, TB69, TB88, TB277, TB173, TB278, TB350, TB349, TB333, TB396 |
| Breast cancer ([7](#_ENREF_7)) | node.positive., hormonal.therapy., TumorGrading., chemotherapy.treatment., Ethnicity., tumor.size..mm.., radiation.treatment., Progesterone.Receptor.status., age.at.diagnosis., EstrogenReceptorStatus. | s0141_hyb, b0341_hyb, s0001_hyb, s1511_hyb, b0556_hyb, s0026_hyb, b0521_hyb, b0433_hyb, s0004_hyb, s0035_hyb, b0383_hyb, s0175_hyb, s0204_hyb, s0071_hyb, b0515_hyb, s0060_hyb, s0037_hyb, s0087_hyb, s0138_hyb, b0428_hyb, s0195_hyb, b0629_hyb, s0168_hyb, s0067_hyb, s0170_hyb, b0427_hyb, b0387_hyb, s0013_hyb, b0305_hyb, b0626_hyb, s0101_hyb, b0251_hyb, s0201_hyb, b0679_hyb, b0394_hyb, b0504_hyb, s0019_hyb, b0326_hyb, s0146_hyb, s0154_hyb, s0046_hyb, b0374_hyb, s0210_hyb, s0080_hyb, s0084_hyb, s0109_hyb, b0667_hyb, b0252_hyb, b0244_hyb |
| Colon cancer ([8](#_ENREF_8)) | Sex, age.at.diagnosis, tnm.t, tnm.n, tnm.m, tumor.location, chemotherapy.adjuvant, mmr.status, cimp.status, cin.status, kras.mutation, braf.mutation | CIT042, CIT063, CIT141, CIT142, CIT160, CIT186, CIT220, CIT305, CIT315, CIT346, CIT393, CIT394, CIT405, CIT410, CIT424, CIT434, CIT438, CIT463, CIT464, CIT488, CIT507, CIT539, CIT548, CIT558, CIT562, CIT027, CIT041, CIT062, CIT071, CIT203, CIT218, CIT233, CIT235, CIT345, CIT352, CIT396, CIT398, CIT404, CIT406, CIT407, CIT408, CIT411, CIT412, CIT413, CIT418, CIT419, CIT420, CIT422, CIT423, CIT439, CIT497, CIT518, CIT545 |
